# Colchicine use and clinical outcomes in acute myocarditis: a retrospective cohort study

**DOI:** 10.3389/fcvm.2026.1930042

**Published:** 2026-10-14

**Authors:** Sukruth Pradeep Kundur, Matthew Sadler, Aamir Shamsi, Cristina Madaudo, William Chick, Daniela Noakes, Mattia Manzoni, Beatrice Perkan, Paul A. Scott, Antonio Cannatà, Daniel I. Bromage

**Affiliations:** 1 School of Cardiovascular Medicine and Sciences, King’s College London British Heart Foundation Centre of Excellence, London, United Kingdom; 2 King’s College Hospital NHS Foundation Trust, London, United Kingdom; 3 Department of Health Promotion, Mother and Child Care, Internal Medicine and Medical Specialties (PROMISE), University of Palermo, Palermo, Italy; 4 Università Degli Studi di Milano, Milan, Italy; 5 KHP Centre for Translational Medicine, London, United Kingdom

**Keywords:** colchicine, heart failure, inflammation, myocarditis, treatment outcome, ventricular dysfunction

## Abstract

**Background:**

Acute myocarditis (AM) is an inflammatory disease of the myocardium associated with ventricular dysfunction, arrhythmia, and progression to cardiomyopathy. Current management remains supportive, and no targeted anti-inflammatory therapy has demonstrated proven benefit on cardiovascular outcomes outside specific histological subtypes. Colchicine is an inexpensive, widely available anti-inflammatory agent with an established role in pericarditis, yet real-world data on its association with clinical outcomes in confirmed AM remain limited.

**Methods:**

Patients with cardiac magnetic resonance-confirmed AM were retrospectively included. The primary outcome was echocardiographic maintenance or recovery of LVEF to a follow-up of ≥50%. Secondary outcomes were all-cause mortality, absolute change in LVEF, and an absolute LVEF improvement of ≥10%.

**Results:**

A total of 243 patients with AM were included in the study, with 63 receiving colchicine and 178 not. Colchicine-treated patients were significantly younger (median 29 vs. 39 years; *p* < 0.001) with a similar sex distribution, although clinical presentation differed between groups. Follow-up LVEF of ≥50%, evaluable in 93 patients, was more frequent in colchicine-treated patients (95% vs. 70%; *p* = 0.035) and persisted after adjustment for age and sex (OR 5.10, 95% CI 1.14–48.7; *p* = 0.03), with directionally consistent results in a propensity score-matched sensitivity analysis. All-cause mortality occurred in 1 of 63 colchicine-treated patients (1.6%) and 12 of 178 untreated patients (6.7%), with no significant difference after age and sex adjustment (OR 0.58, 95% CI 0.06–2.76; *p* = 0.53). Absolute change in LVEF and improvement ≥ 10% did not differ significantly between groups.

**Conclusions:**

Colchicine use was associated with more frequent maintenance or recovery of left ventricular function, although these findings are exploratory in the context of the limitations of this study. Further prospective evidence will highlight the efficacy and scope of colchicine in patients with AM.

## 1 Introduction

Acute myocarditis (AM) is an inflammatory disease of the myocardium that primarily affects young patients in their third/fourth decade of life (1). Clinical presentation is heterogeneous, ranging from subclinical troponin elevation to chest pain, life-threatening ventricular arrhythmias, and cardiogenic shock or death (2). Although most cases have a viral prodrome, the underlying aetiology can be multifactorial with emerging genetic associations with inherited cardiomyopathies (3–5). Prognosis largely depends on the presenting phenotype, with many contributing factors (6). Multicentre registries have reported a higher one-year incidence of cardiac mortality or heart transplantation in patients with a baseline left ventricular ejection fraction (LVEF) below 50%, sustained ventricular arrhythmia, or haemodynamic compromise (1). Cardiac magnetic resonance (CMR) imaging is the preferred non-invasive diagnostic modality and provides both diagnostic and prognostic information, aiding further risk stratification (7, 8). AM is a recognised cause of sudden cardiac death and can progress to non-ischaemic dilated cardiomyopathy (9).

Current management of AM remains largely supportive and is guided by clinical severity and aetiology. The 2025 European Society of Cardiology (ESC) Guidelines for the management of myocarditis and pericarditis recommend symptomatic management, activity restriction, and haemodynamic support where required, while immunosuppression is reserved for specific histological subtypes such as giant-cell or eosinophilic myocarditis (2). The role of disease-modifying pharmacological therapy remains uncertain, with no evidence for a clear benefit on clinical endpoints.

Colchicine is a guideline-recommended standard for the treatment of acute and recurrent pericarditis, including when pericarditis occurs with myocardial involvement (myopericarditis) (10). Its biological rationale extends to isolated myocarditis without pericardial involvement (11), but its role there is not established by guidelines or by dedicated clinical evidence. However, the clinical evidence on the use of colchicine in AM remains limited to administrative database analyses (12) relying on ICD codes, which may be limited by diagnostic misclassification (13). Whether colchicine is associated with improved clinical outcomes or LV function in a cohort of patients with CMR-confirmed AM, therefore, remains unclear. To our knowledge, no previous study has specifically explored the effect of colchicine on left ventricular functional outcomes in patients with acute myocarditis. We aimed to address this gap by examining the association between colchicine use and clinical and echocardiographic patient outcomes. We hypothesised that colchicine use would be associated with improved ventricular maintenance or recovery and lower mortality, consistent with its established anti-inflammatory profile.

## 2 Methods

### 2.1 Study design

We performed a retrospective cohort study of consecutive patients aged 18 years or older with a confirmed diagnosis of AM managed within the cardiology services at King's College Hospital (KCH) and Princess Royal University Hospital (PRUH), United Kingdom. The screening and identification methodology, including patient-level screening and exclusion counts, has been reported in detail for the parent cohort with follow-up to 2022 (6) and as part of a larger registry (14, 15). Consecutive patients admitted between 12 February 2009, and December 2025 were included in this analysis. Patients were identified using CogStack, an open-source clinical data retrieval platform developed at King's College London, with Elasticsearch to interrogate structured and free-text data across hospital databases (16). Additional patients were identified from local Hospital Episode Statistics data where AM was recorded as the primary discharge diagnosis according to the relevant ICD-10 codes (B33.2, I01.2, I09.0, I40, I41, I51.4), and by searching hospital and intensive care unit discharge summaries for patients discharged alive, together with death notifications, as well as those seen in the dedicated Inflammatory Heart Disease clinic at King's College Hospital. Identification was performed iteratively across these sources over the inclusion period.

Patients were eligible if they had a confirmed diagnosis of AM, based on CMR findings consistent with the 2018 updated Lake Louise criteria (17) supported by endomyocardial biopsy according to the Dallas criteria (18) in a proportion of these patients, with available treatment and outcome data. All patients had cardiac magnetic resonance confirmation of AM, and endomyocardial biopsy was additionally performed in 7 patients (2.9%). Diagnostic criteria were applied independently by at least two authors. Inclusion required a clinical presentation consistent with AM and one or more supportive findings on ECG, cardiac biomarkers, or cardiac imaging, in the absence of significant coronary artery disease on invasive or non-invasive coronary imaging, or a low likelihood of coronary artery disease by clinical judgement. Obstructive coronary artery disease was excluded by invasive coronary angiography in 112 patients and by stress perfusion cardiac magnetic resonance in 1 patient. The remaining patients did not have a coronary assessment modality recorded and were adjudicated according to consistency of clinical findings. Patients with COVID-19 or vaccine-related AM (19), cardiac sarcoidosis, or myocarditis secondary to immune checkpoint inhibitor use were excluded, as these represent distinct inflammatory myocardial syndromes with different pathophysiology.

The exposure of interest was colchicine use during the index admission recorded as a binary variable. Dose, timing of initiation, duration of therapy, and continuation after discharge were at the discretion of the prescribing clinician, and data regarding adherence and the clinical indication were not available. Colchicine use in AM is predominantly off-label, with prescribing decisions reflecting individual clinician judgement. Baseline demographic, clinical, echocardiographic, cardiac magnetic resonance, laboratory, treatment, and outcome data were recorded. Follow-up data were extracted from the medical record up to the most recent clinical contact or date of death. The study was conducted under London South-East Research Ethics Committee approval (reference 18/LO/2048) granted to the KERRI (King's Electronic Records Research Interface) and complied with the Declaration of Helsinki (20).

### 2.2 Study outcomes

The primary outcome was maintenance or recovery of left ventricular function, defined as a follow-up echocardiographic left ventricular ejection fraction (LVEF) of ≥50% in patients with available follow-up echocardiography. Continuous LVEF change was not used as the primary outcome because it is subject to a ceiling effect whereby patients with preserved or near-preserved admission LVEF have limited scope for further gain. LVEF ≥50% was therefore used as a dichotomous threshold incorporating the established clinical cut-off for preserved ejection fraction. Secondary outcomes were all-cause mortality, the absolute change in echocardiographic LVEF from admission to last available follow-up, and an absolute LVEF improvement of ≥10%.

### 2.3 Statistical analysis

The results were reported in line with the STROBE (Strengthening the Reporting of Observational Studies in Epidemiology) guidelines (Supplementary Table S1) (21). Continuous variables were expressed as median (Q1-Q3) and categorical variables as counts and percentages. Comparisons between groups were made using the Mann–Whitney *U*-test for continuous variables and Fisher's exact test for categorical variables. Missing data were reported for each variable and were not imputed for further analysis.

For the primary and secondary binary outcomes, inference was based on event counts and Fisher's exact test. Given the limited number of events, adjusted estimates were obtained using Firth penalised logistic regression, which provides finite estimates beyond ordinary logistic regression. Both models were adjusted for age and sex. Given expected sparse events, additional covariates would have exceeded accepted recommendations for events per variable causing issues with model fitting. An exploratory univariable logistic regression was performed to identify clinical predictors of each outcome. Results of logistic and regression models are reported as odds ratios (OR) or coefficients with 95% confidence intervals (CI); continuous variables are summarised as median [interquartile range (IQR)] or mean [standard deviation (SD)] as appropriate.

Subgroup analyses for each outcome were performed across subgroups defined by clinical presentation, admission echocardiographic LVEF, CMR LVEF, and LGE status. Given the small number of evaluable patients and events, the results are reported descriptively.

Propensity score matching was used as a sensitivity analysis rather than as the primary analytical approach, as matching substantially reduced the available sample size and event count. Therefore, the full cohort was retained for the primary analysis to preserve statistical power, with the matched cohort used to assess whether the direction of association was consistent after adjustment for baseline imbalance. The propensity score for colchicine use was estimated using age and sex, with 1:1 nearest-neighbour matching without replacement and a caliper of 0.2 standard deviations of the logit of the propensity score. Covariate balance was assessed using standardised mean differences, with a threshold of 0.1. Additional sensitivity analyses used an expanded propensity score incorporating clinical presentation, admission LVEF, hypertension, CRP and peak troponin, and a further model additionally incorporating cardiac magnetic resonance LVEF. These were applied via 1:1 matching, inverse probability of treatment weighting with stabilised, truncated weights, and inclusion of the logit propensity score as a covariate in the outcome model. Late gadolinium enhancement pattern and NT-proBNP were not included in either model, as these were missing in 28% and 34% of patients, respectively.

A *p*-value of <0.05 was considered significant, with no adjustment for multiple comparisons. All analyses were performed with the R statistical package version 4.5.1 (R Foundation, Vienna, Austria).

## 3 Results

### 3.1 Study population and baseline characteristics

A total of 243 patients with AM were included (Table 1). Median follow-up was 53 (IQR 34–76) months. Approximately two-thirds were male (*n* = 159, 66%) with a median age of 36 years (IQR: 26–51 years). Sixty-three patients (26%) received colchicine, 178 did not, and colchicine status was unavailable for 2 (Figure 1).

**Table 1:** Baseline characteristics of the full cohort.

		Colchicine treatment			
Characteristic	Overall *N* = 241^1^	No colchicine *N* = 178^1^	Colchicine *N* = 63^1^	*p*-value^2^	Missing, *n* (%)
Age (years)	36.0 (26.0, 51.0)	39.0 (27.0, 55.0)	29.0 (23.0, 38.0)	<0.001	0 (0%)
Sex				0.5	0 (0%)
Female	82 (34.0%)	63 (35.4%)	19 (30.2%)		
Male	159 (66.0%)	115 (64.6%)	44 (69.8%)		
Clinical presentation				<0.001	1 (0.4%)
Chest pain	186 (77.5%)	127 (71.8%)	59 (93.7%)		
Heart failure	35 (14.6%)	31 (17.5%)	4 (6.3%)		
Arrhythmia	19 (7.9%)	19 (10.7%)	0 (0.0%)		
NYHA class				0.6	192 (80%)
1	29 (59.2%)	16 (51.6%)	13 (72.2%)		
2	14 (28.6%)	10 (32.3%)	4 (22.2%)		
3	4 (8.2%)	3 (9.7%)	1 (5.6%)		
4	2 (4.1%)	2 (6.5%)	0 (0.0%)		
Chest pain	188 (78.3%)	126 (71.2%)	62 (98.4%)	<0.001	1 (0.4%)
Dyspnoea	79 (33.1%)	56 (31.8%)	23 (36.5%)	0.5	2 (0.8%)
Syncope	5 (2.1%)	4 (2.3%)	1 (1.6%)	>0.9	2 (0.8%)
ST elevation (≥2 mm in ≥2 leads)	64 (30.0%)	42 (26.6%)	22 (40.0%)	0.087	28 (12%)
Hypertension	34 (14.2%)	32 (18.2%)	2 (3.2%)	0.003	2 (0.8%)
Diabetes	14 (5.9%)	13 (7.4%)	1 (1.6%)	0.12	2 (0.8%)
Dyslipidaemia	17 (7.1%)	14 (8.0%)	3 (4.8%)	0.6	2 (0.8%)
Current/ex-smoker	82 (34.2%)	60 (33.9%)	22 (34.9%)	0.9	1 (0.4%)
CMR LV ejection fraction (%)	57.0 (52.0, 63.0)	57.0 (52.0, 64.0)	57.0 (51.0, 62.0)	0.4	8 (3.3%)
CMR LV EDV index (mL/m²)	87.0 (75.0, 98.0)	88.0 (75.0, 100.0)	84.0 (76.0, 94.0)	0.4	53 (22%)
CMR RV ejection fraction (%)	56.0 (52.0, 61.0)	57.0 (52.0, 62.0)	54.0 (51.0, 60.0)	0.11	107 (44%)
Positive T2 mapping	137 (89.0%)	94 (90.4%)	43 (86.0%)	0.4	87 (36%)
LGE pattern				0.069	67 (28%)
0	41 (23.6%)	33 (27.0%)	8 (15.4%)		
1	105 (60.3%)	67 (54.9%)	38 (73.1%)		
2	21 (12.1%)	15 (12.3%)	6 (11.5%)		
3	7 (4.0%)	7 (5.7%)	0 (0.0%)		
Peak troponin (ng/L)	1,642.0 (367.0, 12,753.0)	1,987.5 (312.5, 15,030.5)	1,055.0 (411.0, 3,693.0)	0.3	2 (0.8%)
C-reactive protein (mg/L)	33.0 (7.7, 85.0)	33.0 (7.9, 90.5)	26.0 (7.4, 73.0)	0.8	12 (5.0%)
NT-proBNP (ng/L)	83.0 (60.0, 155.0)	79.0 (60.0, 109.0)	142.0 (72.0, 485.0)	0.024	83 (34%)
White cell count (×10⁹/L)	9.2 (6.7, 12.1)	9.4 (6.7, 12.5)	8.2 (6.9, 11.5)	0.14	3 (1.2%)
Neutrophil-to-lymphocyte ratio	4.1 (2.3, 6.3)	3.7 (2.0, 5.9)	4.2 (2.5, 7.1)	0.10	1 (0.4%)
Admission echo LVEF (%)	55.0 (45.0, 58.0)	54.0 (43.0, 58.0)	55.0 (49.0, 59.0)	0.12	39 (16%)
Preserved (≥50%)	132 (65.3%)	90 (62.1%)	42 (73.7%)		
Mildly reduced (41–49%)	31 (15.3%)	23 (15.9%)	8 (14.0%)		
Moderately reduced (30–40%)	28 (13.9%)	21 (14.5%)	7 (12.3%)		
Severely reduced (<30%)	11 (5.4%)	11 (7.6%)	0 (0.0%)		

Demographic, clinical, echocardiographic, cardiac magnetic resonance, and laboratory characteristics of the 243 patients included with confirmed AM, stratified by colchicine use during the index admission (colchicine, *n* = 63; no colchicine, *n* = 178; colchicine status unavailable, *n* = 2). Continuous variables are presented as median (interquartile range) and compared using the Mann–Whitney *U*-test; categorical variables are presented as number (percentage) and compared using Fisher's exact test. Missing data are reported for each variable where applicable. ACE-I/ARB, angiotensin-converting enzyme inhibitor or angiotensin receptor blocker; NT-proBNP, N-terminal pro-B-type natriuretic peptide; CMR, cardiac magnetic resonance; CRP, C-reactive protein; EMB, endomyocardial biopsy; IQR, interquartile range; LGE, late gadolinium enhancement; LVEF, left ventricular ejection fraction; NYHA, New York Heart Association; RVEF, right ventricular ejection fraction.

1 Median (Q1, Q3); *n* (%).

2 Wilcoxon rank sum test; Fisher's exact test.

**Figure 1:**
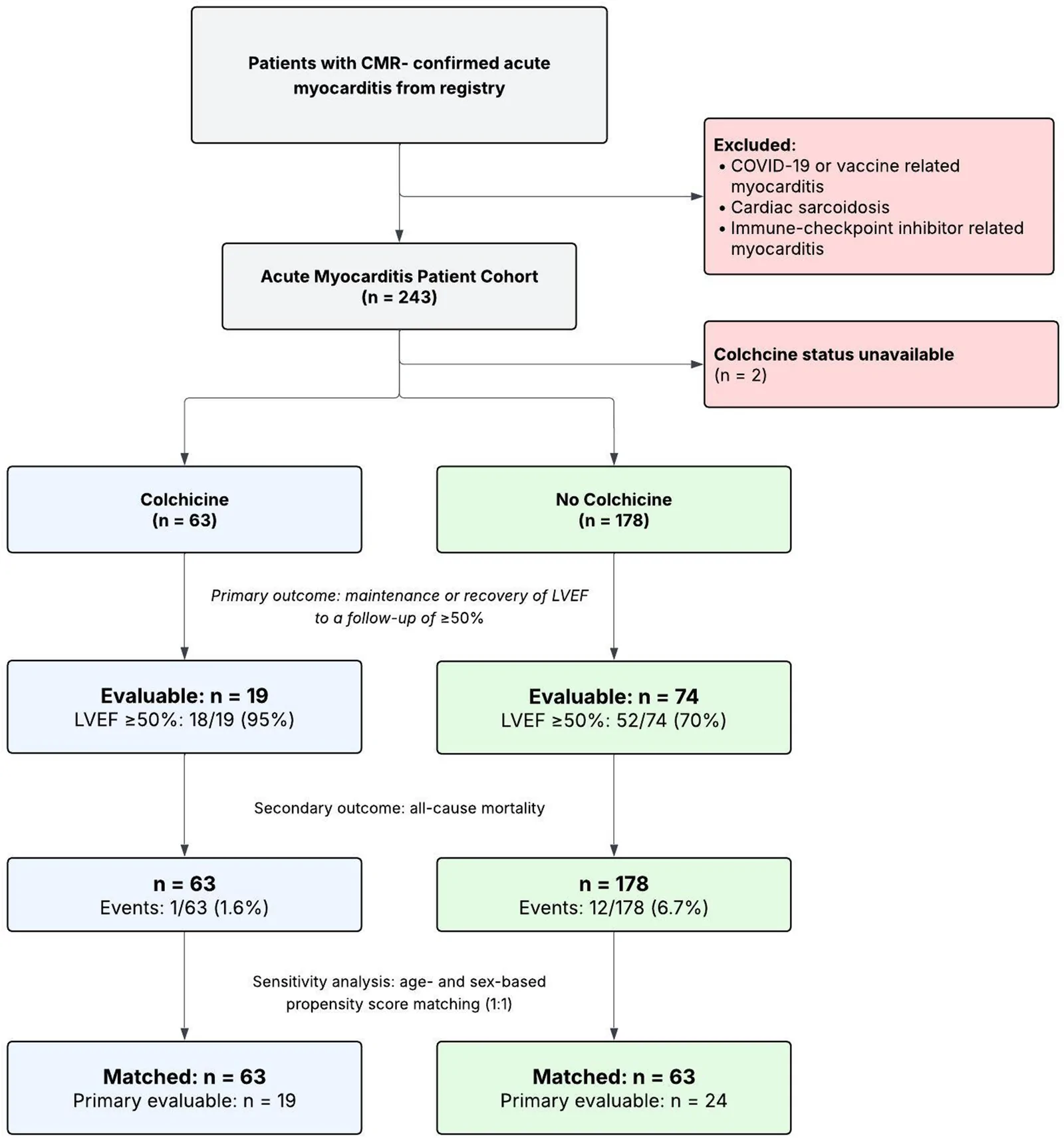
Patient flow diagram. Flow diagram showing patient selection and group allocation. The full cohort included 243 patients with cardiac magnetic resonance-confirmed AM, with endomyocardial biopsy additionally performed in 7 (2.9%). 63 received colchicine during the index admission, and 178 did not. Colchicine status was unavailable for 2 patients.

Patients in the colchicine-treated group were younger [median age 29 years [23–38] vs. 39 years [27–55]; *p* < 0.001], whereas the sex distribution was similar (*p* = 0.50). Clinical presentation differed between groups (*p* < 0.001). An ACS-like presentation was more frequent in colchicine-treated patients (59 of 63, 94%, vs. 127 of 177, 72%), whereas heart failure (4 of 63, 6%, vs. 31 of 178, 18%) and arrhythmia (0 of 63, 0%, vs. 19 of 178, 11%) presentations were more common in untreated patients. Chest pain was almost universal in the colchicine group (62 of 63, 98%, vs. 126 of 177, 71%; *p* < 0.001), while hypertension was less frequent (2 of 63, 3%, vs. 32 of 176, 18%; *p* = 0.003). Diffuse ST elevation was numerically more frequent in colchicine-treated patients (40.0% vs. 26.6%; *p* = 0.087). Baseline ventricular function was comparable between groups, with a median CMR LVEF of 57% in both groups (*p* = 0.4) and a median admission echocardiographic LVEF of 55% vs. 54% (*p* = 0.12). No colchicine-treated patient had severely reduced admission LVEF (<30%), compared with 11 of 145 evaluable untreated patients (7.6%; Table 1). N-terminal pro-B-type natriuretic peptide (NT-proBNP) was higher in colchicine-treated patients (142 vs. 79 ng/L; *p* = 0.02), although this value was missing in a third of the cohort. Concomitant therapy during the index admission is presented in Supplementary Table S2. Aspirin or non-steroidal anti-inflammatory drugs were prescribed in 15 colchicine-treated patients (23.8%) and 68 untreated patients (38.2%; *p* = 0.045), statins in 3 (4.8%) and 37 (20.9%) respectively (*p* = 0.003), immunosuppressive therapy in 4 (6.3%) and 30 (16.9%; *p* = 0.038), and corticosteroids in 2 (3.2%) and 17 (9.7%; *p* = 0.17).

### 3.2 Primary outcome

Follow-up LVEF of ≥50% was evaluable in 93 patients and occurred in 18 of 19 colchicine-treated patients (95%) and in 52 of 74 untreated patients (70%; *p* = 0.035) (Figure 2). The association persisted after adjustment for age and sex using Firth penalised logistic regression (OR 5.10, 95% CI 1.14–48.7; *p* = 0.03) (Table 2, Figure 3). Of the 93 patients with evaluable follow-up echocardiography, 43 (46%) had a preserved admission LVEF (≥50%) and therefore needed only to maintain function, of whom 10 were colchicine-treated and 33 untreated. The remaining 47 (51%) had a reduced admission LVEF (<50%) and required recovery, of whom 8 were colchicine-treated and 39 untreated, with admission LVEF unavailable in 3. Restricted to the 47 patients with a reduced admission LVEF, recovery to ≥50% occurred in 7 of 8 colchicine-treated patients and 22 of 39 untreated patients (unadjusted OR 5.41; *p* = 0.13).

**Figure 2:**
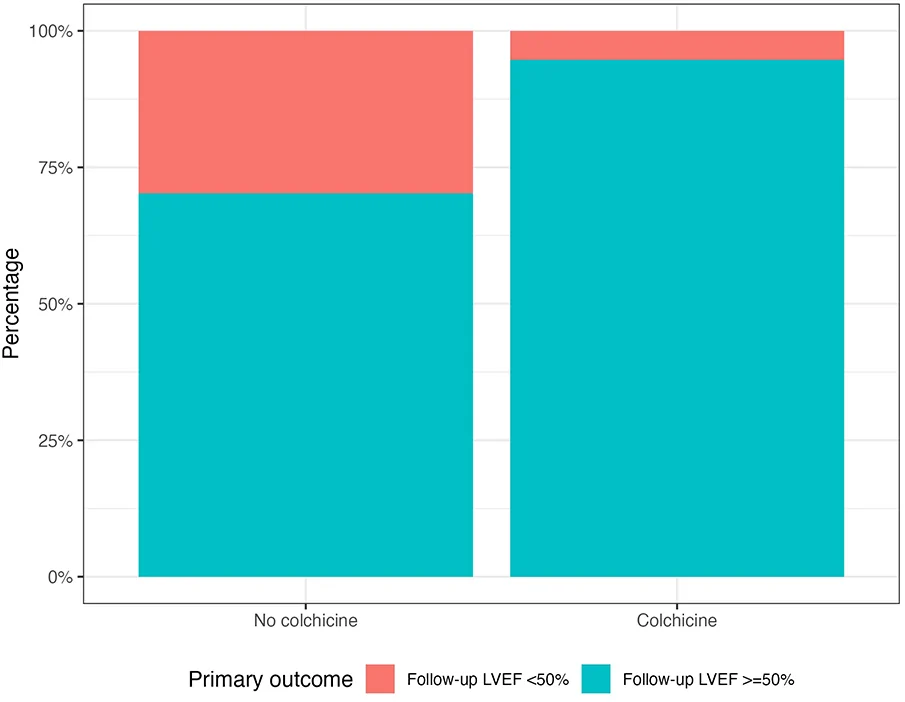
Follow-up LVEF of ≥50% by colchicine treatment (primary outcome). Proportion of patients recovering to a follow-up LVEF of ≥50% among those with available follow-up echocardiography (*n* = 93; colchicine, *n* = 19; no colchicine, *n* = 74). Preserved LV function was observed in 18 of 19 colchicine-treated patients (95%) and 52 of 74 untreated patients (70%; Fisher's exact test *p* = 0.035). Bars show the percentage of patients within each group; teal indicates a follow-up LVEF of ≥50% and red a follow-up LVEF of <50%. LVEF, left ventricular ejection fraction.

**Table 2:** Primary and secondary outcomes.

Outcome	Overall *N* = 241^1^	No colchicine *N* = 178^1^	Colchicine *N* = 63^1^	*p*-value^2^	Missing, *n* (%)
Primary: follow-up LVEF ≥50%				0.035	148 (61%)
Follow-up LVEF <50%	23 (24.7%)	22 (29.7%)	1 (5.3%)		
Follow-up LVEF ≥50%	70 (75.3%)	52 (70.3%)	18 (94.7%)		
Secondary: all-cause mortality				0.19	0 (0%)
Alive	228 (94.6%)	166 (93.3%)	62 (98.4%)		
Death	13 (5.4%)	12 (6.7%)	1 (1.6%)		
Secondary: absolute change in LVEF (%)	3.0 (−2.0, 10.0)	2.5 (−3.0, 10.0)	4.5 (0.0, 10.0)	0.5	151 (63%)
Secondary: LVEF improvement ≥10%				0.6	151 (63%)
<10 percentage point improvement	65 (72.2%)	53 (73.6%)	12 (66.7%)		
≥10 percentage point improvement	25 (27.8%)	19 (26.4%)	6 (33.3%)		

Primary outcome: maintenance or recovery to a follow-up LVEF of ≥50%. Secondary outcomes: all-cause mortality, absolute change in LVEF from admission to follow-up echocardiography, and LVEF improvement of ≥10%. Comparisons were performed using Fisher's exact test for binary outcomes and the Mann–Whitney *U*-test for continuous outcomes. CI, confidence interval; IQR, interquartile range; LVEF, left ventricular ejection fraction; OR, odds ratio.

1 *n* (%); Median (Q1, Q3).

2 Fisher's exact test; Wilcoxon rank sum test.

**Figure 3:**
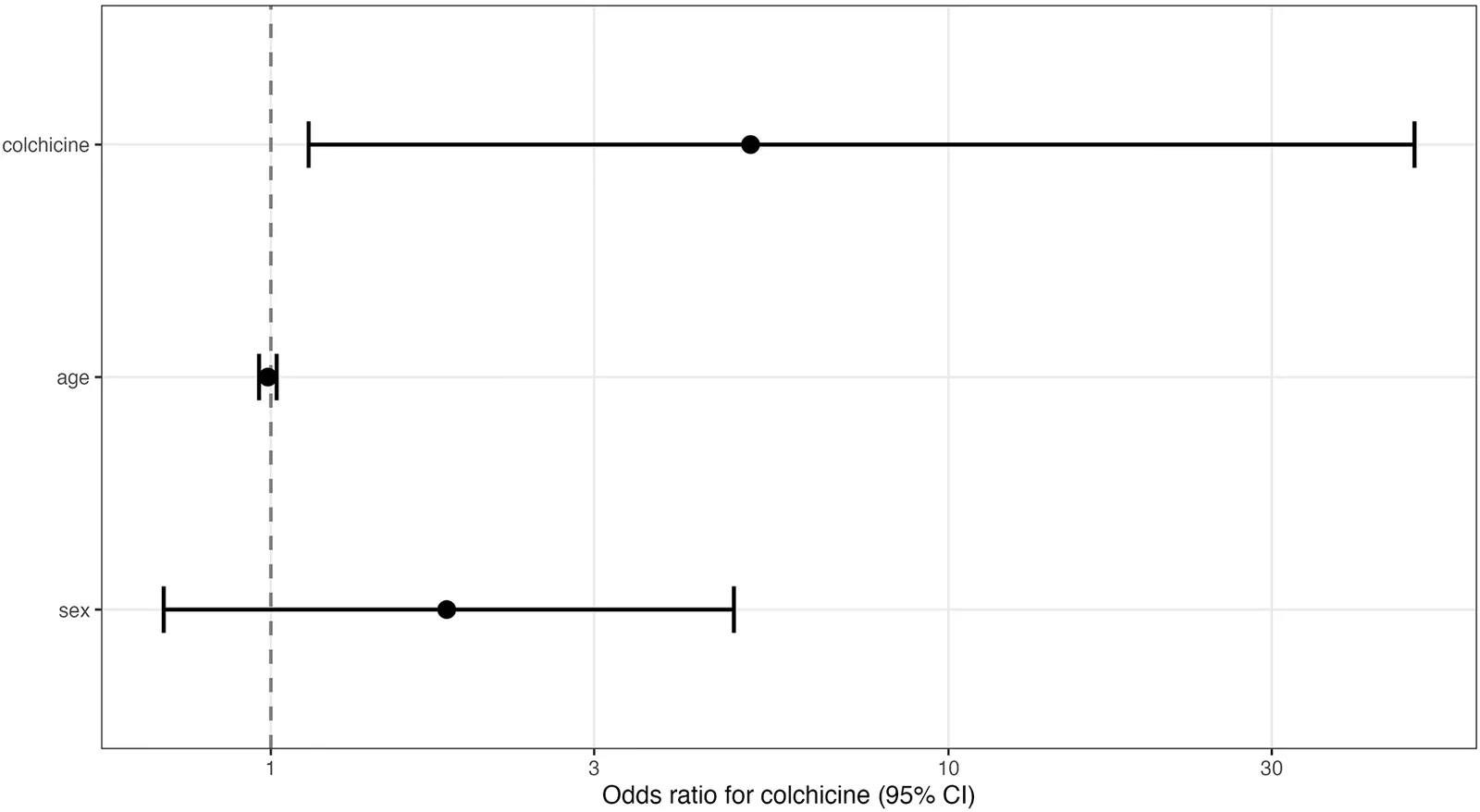
Age- and sex-adjusted treatment effect for the primary outcome. Forest plot of odds ratios with 95% confidence intervals from Firth penalised logistic regression for follow-up LVEF of ≥50% (*n* = 93; colchicine, *n* = 19; no colchicine, *n* = 74), adjusted for colchicine treatment, age, and sex. Points represent odds ratio estimates; horizontal lines represent 95% confidence intervals; the vertical dashed line represents an odds ratio of 1.0. Odds ratios are plotted on a log scale. CI, confidence interval; LVEF, left ventricular ejection fraction.

### 3.3 Secondary outcomes

All-cause mortality was evaluable in 241 patients and occurred in 1 of 63 colchicine-treated patients (1.6%) and in 12 of 178 untreated patients (6.7%; OR 0.22, 95% CI 0.005–1.57; *p* = 0.19). With the sparse events, this cohort was underpowered to detect any change in mortality. In the Firth penalised logistic regression model adjusted for age and sex, the association of colchicine with mortality was not statistically significant (OR 0.58, 95% CI 0.06–2.76; *p* = 0.53), with wide confidence intervals reflecting the single event in the colchicine arm (Figure 4). Age was independently associated with mortality (OR 1.06 per year, 95% CI 1.02–1.10; *p* = 0.002) and univariable analysis identified arrhythmic presentation (OR 8.00, 95% CI 1.88–31.3; *p* = 0.003), hypertension (OR 8.60, 95% CI 2.67–28.6; *p* < 0.001), and diabetes (OR 15.1, 95% CI 3.92–55.6; *p* < 0.001) as each associated with all-cause mortality (Supplementary Table S3), consistent with previous studies (1).

**Figure 4:**
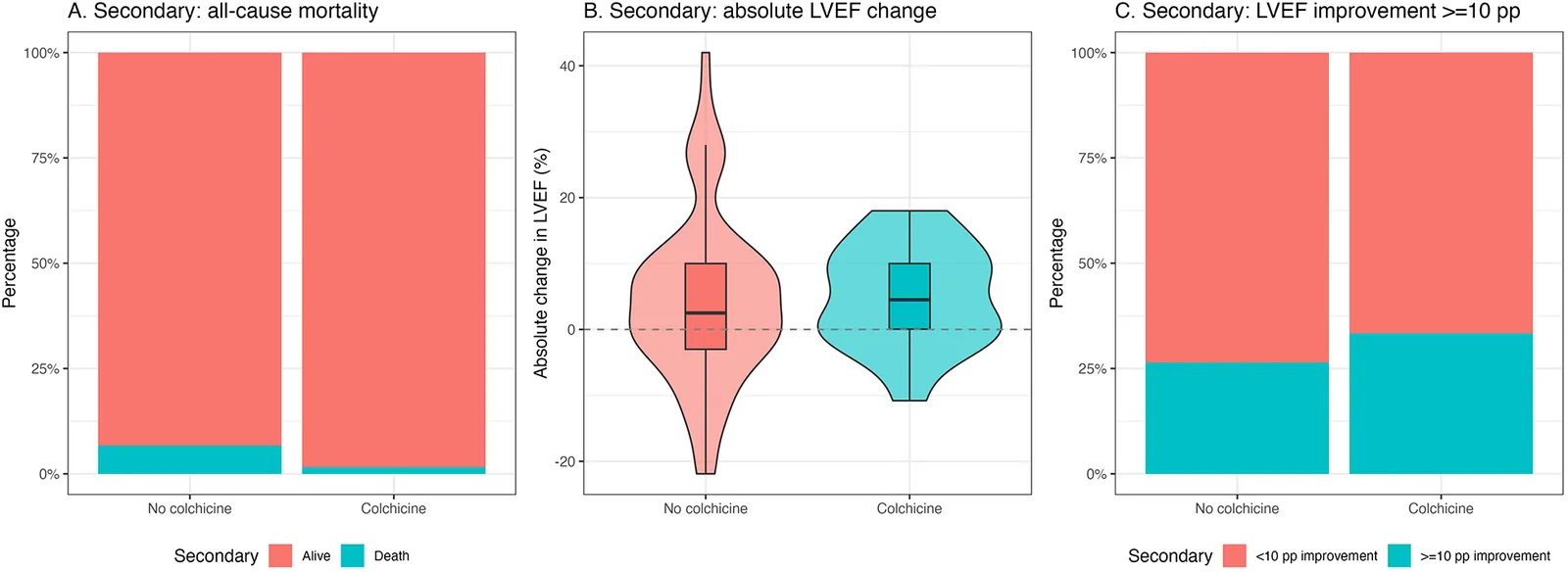
Secondary outcomes by colchicine treatment. **(A)** Shows the proportion of patients who died (*n* = 241; colchicine, *n* = 63; no colchicine, *n* = 178). **(B)** Shows the distribution of the absolute change in echocardiographic LVEF from admission to last available follow-up, among patients with paired measurements (*n* = 90; colchicine, *n* = 18; no colchicine, *n* = 72); boxes represent the median and interquartile range, whiskers the range, and the horizontal dashed line no change. **(C)** Shows the proportion of patients with an absolute LVEF improvement of ≥10% (*n* = 90; colchicine, *n* = 18; no colchicine, *n* = 72). Binary outcomes were compared using Fisher's exact test, and the continuous outcome was compared using the Mann–Whitney *U*-test. LVEF, left ventricular ejection fraction.

The median change in LVEF from admission to the last available echocardiography was 4.5 percentage points (IQR 0.0–10.0) in colchicine-treated patients compared with 2.5 percentage points (IQR −3.0 to 10.0) in untreated patients (Figure 4). In linear regression adjusted for admission LVEF, age, and sex, colchicine was not independently associated with LVEF change (*β* = 2.7, 95% CI −1.6 to 7.1; *p* = 0.22), whereas lower LVEF on admission was the dominant predictor of LVEF change (*β* = −0.71, 95% CI −0.85 to −0.57; *p* < 0.001). Absolute LVEF improvement of ≥10% did not differ significantly between groups (33% vs. 26%; adjusted OR 1.54, 95% CI 0.49–4.64; *p* = 0.45).

### 3.4 Propensity score matched sensitivity analysis

Age- and sex-based propensity score matching resulted in a matched cohort of 126 patients (63 per group), with all standardised mean differences below 0.1 after matching (Supplementary Table S4, Supplementary Figure S1). Matched analyses were performed for both binary outcomes (primary outcome and all-cause mortality). In the matched cohort, follow-up LVEF of ≥50% occurred in 18 of 19 colchicine-treated patients (95%) and in 17 of 24 untreated patients (71%; OR 6.55, 95% CI 1.14–74.7; *p* = 0.03) (Supplementary Table S5). Furthermore, all-cause mortality occurred in 1 of 63 colchicine-treated patients (1.6%) and in 2 of 63 untreated patients (3.2%; OR 0.61, 95% CI 0.06–4.67; *p* = 0.63). The direction of association for both outcomes, when adjusting for age and sex, was consistent with the full-cohort analysis. In sensitivity analyses using the expanded propensity score (Supplementary Figure S2), point estimates for LVEF recovery remained in a similar range to the primary analysis across both covariate sets and all three methods (OR 3.89–5.00). Statistical significance was inconsistent as propensity-score-covariate-adjusted regression reached the conventional threshold for both the seven-covariate model (OR 4.67, 95% CI 1.03–44.73, *p* = 0.046) and the eight-covariate model additionally including CMR LVEF (OR 4.86, 95% CI 1.07–46.53, *p* = 0.040), whereas matching and inverse probability weighting did not reach significance under either covariate set (Supplementary Table S8), reflecting a small remaining evaluable sample once follow-up echocardiography availability was combined with matching or weighting.

## 4 Discussion

By evaluating colchicine use in a clinically characterised cohort of patients with CMR- or EMB-confirmed AM, incorporating patient-level imaging and echocardiographic follow-up data, this study extends the emerging evidence base (10, 12). In this cohort, among patients with available follow-up echocardiography, colchicine use was associated with more frequent preserved left ventricular systolic function. All-cause mortality did not differ significantly between groups, with the single event in the colchicine arm precluding any comparison.

### 4.1 Colchicine in AM

Colchicine is a widely available anti-inflammatory agent with an established safety profile routinely used across many cardiovascular and rheumatological indications. Specifically in pericarditis, the COPE (22), CORE (23), CORP (24), and ICAP trials (25) established the efficacy of colchicine in reducing the recurrence of pericarditis in addition to standard anti-inflammatory therapy. More widely, colchicine has shown significant reductions in atherothrombotic events in the COLCOT trial among patients following myocardial infarction (26) and in the LoDoCo2 trial among patients with chronic stable coronary artery disease (27). In the 2025 ESC guidelines, colchicine is assigned a Class IIa recommendation in the treatment of myocarditis when there is pericarditis with concomitant myocardial involvement (2).

In the context of myocarditis, there is a biological rationale for colchicine therapy (11). Mechanistically, colchicine disrupts microtubule polymerisation and impairs NLRP3 inflammasome assembly (28), reducing downstream IL-1*β* and IL-18-mediated inflammation (29). Both processes are particularly relevant in AM, where innate immune activation and inflammatory cell recruitment contribute to the ongoing myocardial injury with direct implications for ventricular function (30). In a murine model of coxsackievirus B3 myocarditis, colchicine improved left ventricular function and reduced NLRP3 inflammasome activity without impairing viral clearance (31). Equally, AM is thought to be driven largely by an adaptive CD4+ Th1/Th17 response, which colchicine does not directly target (28). An effect is therefore more likely to act on the innate inflammasome pathway rather than the immune trigger.

Clinically, our findings are directionally consistent with emerging observational evidence. In a large TriNetX real-world analysis of adults hospitalised with AM, Golino et al. reported lower 90-day rates of all-cause death, ventricular arrhythmias, and acute heart failure among colchicine-treated patients after propensity score matching (HR 0.66; 95% CI 0.55–0.79; *p* < 0.001) (12). However, that analysis relied on administrative ICD codes, and it was also limited by incomplete treatment information. In patients with myopericarditis, Collini et al. similarly found fewer recurrences after colchicine treatment (*n* = 175; 73 matched pairs), with recurrence rates of 19.2% vs. 43.8% after propensity score matching and an adjusted HR of 0.39 (95% CI 0.21–0.76; *p* = 0.005) (10).

### 4.2 Interpretation of clinical findings

The result for the primary outcome showed more frequent LVEF≥50% among patients with available follow-up echocardiography. However, this estimate should be interpreted as exploratory, with the wide confidence interval reflecting the small number of colchicine-treated patients with evaluable follow-up echocardiography (*n* = 19, of whom 18 recovered). Nevertheless, this is consistent with the mechanistic theory that if colchicine attenuates NLRP3-mediated myocardial inflammation, its most detectable effect in a predominantly low-risk cohort would be on ventricular remodelling rather than mortality. The lack of a significant difference in absolute LVEF improvement of ≥10% and linear regression identifying lower admission LVEF as the dominant predictor of LVEF change support this, as the preserved baseline LVEF in most patients could limit the scope for large absolute gains. Notably, effect estimates were imprecise after Firth regression, with wide confidence intervals reflecting the limited number of evaluable patients.

All-cause mortality did not differ significantly between groups (1.6% vs. 6.7%; *p* = 0.19). These low event rates are consistent with the epidemiology of contemporary AM cohorts in which hard clinical endpoints are uncommon. In the Lombardy registry, cardiac mortality or transplantation occurred in only 3.0% of patients at one year, with substantially higher rates only in those with complicated presentations (1). The scarcity of hard events in our cohort reflects this phenotype, and it limited the power of the mortality analyses.

The results of this study are interpreted cautiously as hypothesis-generating. Colchicine-treated patients were younger, more frequently presented with chest pain, and had a lower comorbidity burden. The associations of arrhythmic presentation, hypertension, and diabetes with all-cause mortality suggest that the higher-risk phenotype was underrepresented in the colchicine arm. The consistency of directional findings after propensity score matching for age and sex provides reassurance, but confounding by presenting phenotype cannot be excluded (Supplementary Table S6).

There is also a possible effect attributable to concurrent pericarditis involvement, since colchicine carries a Class IIa ESC 2025 guideline recommendation specifically in the context of myocarditis with concomitant pericarditis (2). Systematic data on pericardial involvement were not available in this cohort, and its contribution to the observed association cannot be quantified. As this study was conducted across two large tertiary centres with ethnically diverse patient populations and a dedicated cardiology service, generalisability remains relevant to similar centres, although further prospective multi-centre validation is necessary. Because systematic pericardial assessment was not available, it is not possible to determine what proportion of the colchicine-treated group had clinical or imaging evidence of pericardial involvement. Given that colchicine already carries a guideline indication in myopericarditis, and that treated patients had more frequent chest pain and diffuse ST elevation, we cannot rule out confounding by indication.

### 4.3 The evolving trial landscape

These findings should be interpreted in the context of the ongoing studies evaluating anti-inflammatory therapy in inflammatory myocardial disease. The Myocarditis Treatment Trial remains the only adequately powered prospective randomised study of immunosuppression in any myocarditis patients (32). However, this study found no improvement in survival or LVEF recovery with prednisone and cyclosporine or azathioprine compared with standard therapy.

There are two ongoing prospective randomised controlled trials specifically evaluating colchicine across the spectrum of myocardial inflammation. The ARGO trial (NCT05855746) is a phase III, multicentre, double-blind study randomising patients hospitalised with AM to three months of colchicine or placebo in addition to standard care, with late gadolinium enhancement mass on CMR and clinical outcomes as endpoints. The CMP-MYTHiC trial (NCT06158698) is evaluating colchicine in chronic inflammatory cardiomyopathy and incorporates an associated registry assessing the prognostic value of pathogenic genetic variants (33). Together, these trials incorporate the acute and chronic phases of inflammatory myocardial disease and reflect the growing recognition of the overlap between myocarditis and genetic cardiomyopathies (34, 35). Our analysis contributes hypothesis-generating, patient-level data from a cohort with AM confirmed by validated diagnostic criteria to the evolving literature.

Further context for the difficulties of evaluating anti-inflammatory therapies in myocarditis is provided by the phase IIb ARAMIS trial, in which 120 patients with CMR-proven AM received the IL-1 receptor antagonist anakinra or placebo within 72 h of admission until discharge, in addition to standard care (36). The study was neutral on its primary composite endpoint, in part reflecting the low-risk profile of the enrolled population and lack of confirmation of cases (37). With a scarcity of clinical endpoints, our cohort supports these suggestions, indicating that studies may need to recruit high-risk cohorts to evaluate survival benefits in AM. One practical approach to such enrichment is risk stratification using widely available biomarkers (38). With a larger cohort of the same registry, an elevated neutrophil-to-lymphocyte ratio was associated with adverse outcomes across the left ventricular ejection fraction spectrum in AM (14, 15). Risk stratification for trial enrichment by clinical parameters is further supported by the Re-Myo study's identification of family history of cardiomyopathy, autoimmune disease, and ring-like LGE as independent predictors of recurrent AM (39).

## 5 Limitations

This study has an observational retrospective design; consequently, the results are limited to exploratory analysis rather than causal inference. The primary outcome combines maintenance of preserved ventricular function with recovery from impaired function, which may reflect different underlying processes. The present cohort was too small to analyse these separately with adequate precision. Furthermore, the primary outcome was evaluable in only 93 of 243 patients, with follow-up echocardiography missing in 70% of colchicine-treated patients and 58% of untreated patients. Missingness was associated with baseline disease severity, as patients with evaluable follow-up echocardiography had a lower admission LVEF (median 47.5% vs. 55.0%) and more frequently presented with heart failure than those without (Supplementary Table S7). These data may therefore not be missing at random. The interval between the index admission and follow-up echocardiography was not recorded, and follow-up imaging was obtained at clinical discretion rather than at a protocol-driven timepoint. Colchicine was prescribed at the discretion of the treating clinician and concomitant therapy also differed between groups, with untreated patients more often receiving aspirin or non-steroidal anti-inflammatory drugs, statins, immunosuppressive therapy and corticosteroids (Supplementary Table S2). Data on findings such as pericardial effusion, pericarditic chest pain, and CMR pericardial enhancement suggestive of concurrent pericarditis were not available. Additionally, the study was not powered to assess mortality, ventricular arrhythmia, heart failure hospitalisation, or any other hard clinical outcome.

## 6 Conclusion

Colchicine is a well-established, inexpensive, and widely available anti-inflammatory therapy with biological plausibility and hypothesis-generating evidence that it may be associated with more preserved left ventricular function in patients with AM. In the context of ongoing studies, this study provides further data supporting the prospective evaluation of colchicine in AM.

## Data Availability

The datasets presented in this article are not readily available because the data are not publicly available, comprising patient-level clinical records collected under the King's Electronic Records Research Interface approval (London South-East Research Ethics Committee reference 18/LO/2048), which does not permit public sharing. Anonymised data may be available from the corresponding author on reasonable request, subject to the necessary approvals and a data sharing agreement. Requests to access the datasets should be directed to daniel.bromage@kcl.ac.uk.
