# Supplementary material for: Colchicine use and clinical outcomes in acute myocarditis: a retrospective cohort study
Source: Front Cardiovasc Med. 2026 Oct 14;13:1930042. doi: 10.3389/fcvm.2026.1930042 (PMC13713774; doi:10.3389/fcvm.2026.1930042)
Supplement: Supplementary file 1 [file Datasheet1.docx]

**Supplementary Material**

Colchicine Use and Clinical Outcomes in Acute Myocarditis: A Retrospective Cohort Study

**Contents**

**SUPPLEMENTARY FIGURES**

Supplementary Figure 1 - Covariate balance before and after propensity score matching for age and sex.

Supplementary Figure 2 - Covariate balance before and after propensity score matching using the expanded propensity score model.

**SUPPLEMENTARY TABLES**

Supplementary Table 1 – STROBE checklist

Supplementary Table 2 – Concomitant therapy by treatment group

Supplementary Table 3 – Univariable predictors of all-cause mortality

Supplementary Table 4 – Baseline characteristics of propensity score-matched cohort

Supplementary Table 5 – Clinical outcomes in propensity score-matched cohort

Supplementary Table 6 – Baseline characteristics for the propensity score-matched cohort using an expanded covariate set

Supplementary Table 7 – Sensitivity analysis of patients with and without follow-up echocardiography

Supplementary Table 8 - Sensitivity analyses of the colchicine treatment effect for LVEF recovery to ≥50% using expanded propensity score models.

# Supplementary Figures

**Supplementary Figure 1**

**Supplementary Figure 1. Covariate balance before and after propensity score matching for age and sex.**

Plot showing standardised mean differences for age, sex, and the propensity score distance in the full unmatched cohort (unadjusted; salmon) and the 1:1 nearest-neighbour propensity score matched cohort (adjusted; teal). The solid vertical line represents an SMD of 0.0; the dashed vertical line represents the balance threshold of SMD=0.1. After matching, variables achieved SMDs below 0.1 (age SMD=0.007 and sex SMD=0.034; propensity score SMD=0.006).

**PSM,** propensity score matching; **SMD,** standardised mean difference.

**Supplementary Figure 2**

**Supplementary Figure 2. Covariate balance before and after propensity score matching using the expanded propensity score model.**

# Absolute standardised mean differences (SMD) before (unadjusted; salmon) and after (adjusted; teal) 1:1 nearest-neighbour propensity score matching using the expanded eight-covariate model (age, sex, clinical presentation, admission echocardiographic LVEF, hypertension, log CRP, log peak troponin and CMR LVEF), with matching performed among patients with complete data on all eight covariates (caliper 0.2 SD of the logit propensity score; 106 matched patients, 53 per group). The solid vertical line represents an SMD of 0.0; the dashed vertical line represents the balance threshold of SMD=0.1. "Distance" denotes the propensity score. Residual imbalance above 0.1 remained after matching for some covariates, most notably log peak troponin and admission echocardiographic LVEF. CMR, cardiac magnetic resonance; CRP, C-reactive protein; LVEF, left ventricular ejection fraction; SMD, standardised mean difference.

# Supplementary Tables

## Supplementary Table 1

| **Section / Item** | **No.** | **Recommendation** | **Location in manuscript** |
| --- | --- | --- | --- |
| **TITLE AND ABSTRACT** | | | |
| Title and abstract | 1a | (a) Indicate the study's design with a commonly used term in the title or the abstract | Title; Abstract Methods |
|  | 1b | (b) Provide in the abstract an informative and balanced summary of what was done and what was found | Abstract: all four structured sections (Background, Methods, Results, Conclusions) |
| **INTRODUCTION** | | | |
| Background / rationale | 2 | Explain the scientific background and rationale for the investigation being reported | Introduction paragraphs 1–2 |
| Objectives | 3 | State specific objectives, including any prespecified hypotheses | Introduction, final paragraph |
| **METHODS** | | | |
| Study design | 4 | Present key elements of study design early in the paper | Methods - Study Design |
| Setting | 5 | Describe the setting, locations, and relevant dates, including periods of recruitment, exposure, follow-up, and data collection | Methods - Study Design |
| Participants | 6a | (a) Give the eligibility criteria, and the sources and methods of selection of participants. Describe methods of follow-up | Methods - Study Design |
|  | 6b | (b) For matched studies, give matching criteria and number of exposed and unexposed | Methods – Statistical Analysis |
| Variables | 7 | Clearly define all outcomes, exposures, predictors, potential confounders, and effect modifiers. Give diagnostic criteria, if applicable | Methods - Study Outcomes |
| Data sources / measurement | 8* | For each variable of interest, give sources of data and details of methods of assessment (measurement). Describe comparability of assessment methods if there is more than one group | Methods - Study Design |
| Bias | 9 | Describe any efforts to address potential sources of bias | Methods - Statistical Analysis |
| Study size | 10 | Explain how the study size was arrived at | Methods - Study Design |
| Quantitative variables | 11 | Explain how quantitative variables were handled in the analyses. If applicable, describe which groupings were chosen and why | Methods - Study Outcomes |
| Statistical methods | 12a | (a) Describe all statistical methods, including those used to control for confounding | Methods - Statistical Analysis |
|  | 12b | (b) Describe any methods used to examine subgroups and interactions | Methods - Statistical Analysis |
|  | 12c | (c) Explain how missing data were addressed | Methods - Statistical Analysis |
|  | 12d | (d) If applicable, explain how loss to follow-up was addressed | Methods - Study Outcomes |
|  | 12e | (e) Describe any sensitivity analyses | Methods - Statistical Analysis |
| **RESULTS** | | | |
| Participants | 13a | (a) Report numbers of individuals at each stage of study—eg numbers potentially eligible, examined for eligibility, confirmed eligible, included in the study, completing follow-up, and analysed | Results - Study Population; Figure 1 |
|  | 13b | (b) Give reasons for non-participation at each stage | Results - Study Population; Figure 1 |
|  | 13c | (c) Consider use of a flow diagram | Figure 1 |
| Descriptive data | 14a | (a) Give characteristics of study participants (eg demographic, clinical, social) and information on exposures and potential confounders | Table 1 |
|  | 14b | (b) Indicate number of participants with missing data for each variable of interest | Table 1 |
|  | 14c | (c) Summarise follow-up time (eg, average and total amount) | Results - Study Population |
| Outcome data | 15* | Report numbers of outcome events or summary measures over time | Results – Primary Outcome; Secondary Outcomes; Limitations |
| Main results | 16a | (a) Give unadjusted estimates and, if applicable, confounder-adjusted estimates and their precision (eg, 95% confidence interval). Make clear which confounders were adjusted for and why they were included | Results – Primary Outcome; Secondary Outcomes |
|  | 16b | (b) Report category boundaries when continuous variables were categorized | Methods - Study Outcomes |
|  | 16c | (c) If relevant, consider translating estimates of relative risk into absolute risk for a meaningful time period | N/A |
| Other analyses | 17 | Report other analyses done—eg analyses of subgroups and interactions, and sensitivity analyses | Results |
| **DISCUSSION** | | | |
| Key results | 18 | Summarise key results with reference to study objectives | Discussion first section |
| Limitations | 19 | Discuss limitations of the study, taking into account sources of potential bias or imprecision. Discuss both direction and magnitude of any potential bias | Limitations |
| Interpretation | 20 | Give a cautious overall interpretation of results considering objectives, limitations, multiplicity of analyses, results from similar studies, and other relevant evidence | Discussion |
| Generalisability | 21 | Discuss the generalisability (external validity) of the study results | Discussion - Interpretation of clinical findings |
| **OTHER INFORMATION** | | | |
| Funding | 22 | Give the source of funding and the role of the funders for the present study and, if applicable, for the original study on which the present article is based | Funding |

**Supplementary Table 1. Completed STROBE checklist.**

The completed Strengthening the Reporting of Observational Studies in Epidemiology (STROBE) checklist for cohort studies, indicating the location within the manuscript where each required item has been addressed.

**STROBE,** Strengthening the Reporting of Observational Studies in Epidemiology.

## Supplementary Table 2. Concomitant therapy by treatment group

| **Concomitant therapy** | **Overall**  N = 241^1^ | **No colchicine**  N = 178^1^ | **Colchicine**  N = 63^1^ | **p-value**^2^ |
| --- | --- | --- | --- | --- |
| **Aspirin or NSAID** | 83 (34.4%) | 68 (38.2%) | 15 (23.8%) | 0.045 |
| **Corticosteroid** | 19 (8.0%) | 17 (9.7%) | 2 (3.2%) | 0.2 |
| Missing | 3 | 3 | 0 |  |
| **Immunosuppressant** | 34 (14.2%) | 30 (16.9%) | 4 (6.3%) | 0.038 |
| Missing | 1 | 1 | 0 |  |
| **Statin** | 40 (16.7%) | 37 (20.9%) | 3 (4.8%) | 0.003 |
| Missing | 1 | 1 | 0 |  |
| **RAAS inhibitor** | 115 (47.7%) | 90 (50.6%) | 25 (39.7%) | 0.15 |
| **Beta-blocker** | 117 (48.5%) | 91 (51.1%) | 26 (41.3%) | 0.2 |
| **Mineralocorticoid receptor antagonist** | 15 (6.3%) | 13 (7.3%) | 2 (3.2%) | 0.4 |
| Missing | 1 | 0 | 1 |  |
| **Diuretic** | 22 (9.1%) | 17 (9.6%) | 5 (7.9%) | 0.8 |
| **Amiodarone** | 4 (1.7%) | 4 (2.2%) | 0 (0.0%) | 0.6 |
| **Anticoagulant (any)** | 24 (10.0%) | 22 (12.4%) | 2 (3.2%) | 0.048 |
| **Inotrope** | 23 (9.7%) | 18 (10.3%) | 5 (7.9%) | 0.8 |
| Missing | 3 | 3 | 0 |  |
| **Renal replacement therapy** | 15 (6.3%) | 13 (7.4%) | 2 (3.2%) | 0.4 |
| Missing | 3 | 3 | 0 |  |
| ^1^n (%) | | | | |
| ^2^Fisher's exact test | | | | |

## Concomitant pharmacological therapy prescribed during the index admission, by colchicine treatment status. NSAID, non-steroidal anti-inflammatory drug; RAAS, renin–angiotensin–aldosterone system.

**Supplementary Table 3. Univariable predictors of all-cause mortality.**

| **Characteristic** | **N** | **OR** | **95% CI** | **p-value** |
| --- | --- | --- | --- | --- |
| **Colchicine treatment** | 241 | 0.22 | 0.01, 1.17 | 0.2 |
| **Age (years)** | 241 | 1.06 | 1.02, 1.10 | **0.002** |
| **Male sex** | 241 | 0.82 | 0.26, 2.78 | 0.7 |
| **Clinical presentation** | 240 |  |  |  |
| ACS-like (reference) |  | — | — |  |
| Heart failure |  | 2.81 | 0.57, 11.2 | 0.2 |
| Arrhythmia |  | 8.00 | 1.88, 31.3 | **0.003** |
| **NYHA class** | 49 | 3.53 | 1.09, 14.6 | **0.042** |
| **Hypertension** | 239 | 8.60 | 2.67, 28.6 | **<0.001** |
| **Diabetes** | 239 | 15.1 | 3.92, 55.6 | **<0.001** |
| **CMR LVEF (%)** | 233 | 1.02 | 0.96, 1.09 | 0.6 |
| **CMR RVEF (%)** | 134 | 0.95 | 0.85, 1.07 | 0.4 |
| **Positive T2 mapping** | 154 | 1.26 | 0.22, 23.9 | 0.8 |
| **LGE pattern** | 174 |  |  |  |
| 0 |  | — | — |  |
| 1 |  | 0.29 | 0.07, 1.13 | 0.072 |
| 2 |  | 1.20 | 0.23, 5.46 | 0.8 |
| 3 |  | 1.20 | 0.06, 9.40 | 0.9 |
| **Peak troponin** | 239 | 1.00 | 1.00, 1.00 | 0.6 |
| **CRP (mg/L)** | 229 | 1.00 | 0.99, 1.01 | >0.9 |
| **NT-proBNP (ng/L)** | 158 | 1.00 | 1.00, 1.00 | 0.2 |
| Abbreviations: CI = Confidence Interval, OR = Odds Ratio | | | | |

Results from individual univariable logistic regression models for each predictor of all-cause mortality in 241 patients (13 events). For clinical presentation, ACS-like presentation is the reference category. The colchicine estimate is from univariable logistic regression and therefore differs slightly from the Fisher's exact test estimate reported in the main text.

**ACS,** acute coronary syndrome; **CI,** confidence interval; **CMR,** cardiac magnetic resonance; **CRP,** C-reactive protein; **LGE,** late gadolinium enhancement; **LVEF,** left ventricular ejection fraction; **NT-proBNP,** N-terminal pro-B-type natriuretic peptide; **NYHA,** New York Heart Association; **OR,** odds ratio; **RVEF,** right ventricular ejection fraction.

**Supplementary Table 4. Baseline characteristics of the propensity score matched cohort.**

| **Characteristic** | **Overall**  N = 126^1^ | **No colchicine**  N = 63^1^ | **Colchicine**  N = 63^1^ | **p-value**^2^ |
| --- | --- | --- | --- | --- |
| **Age (years)** | 29.0 (23.0, 38.0) | 29.0 (23.0, 38.0) | 29.0 (23.0, 38.0) | >0.9 |
| **Sex** |  |  |  | >0.9 |
| Female | 39 (31.0%) | 20 (31.7%) | 19 (30.2%) |  |
| Male | 87 (69.0%) | 43 (68.3%) | 44 (69.8%) |  |
| **Clinical presentation** |  |  |  | **0.034** |
| ACS-like | 110 (87.3%) | 51 (81.0%) | 59 (93.7%) |  |
| Heart failure | 11 (8.7%) | 7 (11.1%) | 4 (6.3%) |  |
| Arrhythmia | 5 (4.0%) | 5 (7.9%) | 0 (0.0%) |  |
| **NYHA class** |  |  |  | 0.7 |
| 1 | 20 (66.7%) | 7 (58.3%) | 13 (72.2%) |  |
| 2 | 7 (23.3%) | 3 (25.0%) | 4 (22.2%) |  |
| 3 | 2 (6.7%) | 1 (8.3%) | 1 (5.6%) |  |
| 4 | 1 (3.3%) | 1 (8.3%) | 0 (0.0%) |  |
| Missing | 96 | 51 | 45 |  |
| **Chest pain** | 113 (90.4%) | 51 (82.3%) | 62 (98.4%) | **0.002** |
| Missing | 1 | 1 | 0 |  |
| **Dyspnoea** | 40 (32.0%) | 17 (27.4%) | 23 (36.5%) | 0.3 |
| Missing | 1 | 1 | 0 |  |
| **Syncope** | 1 (0.8%) | 0 (0.0%) | 1 (1.6%) | >0.9 |
| Missing | 1 | 1 | 0 |  |
| **ST elevation (≥2 mm in ≥2 leads)** | 39 (34.8%) | 17 (29.8%) | 22 (40.0%) | 0.3 |
| Missing | 14 | 6 | 8 |  |
| **Hypertension** | 8 (6.4%) | 6 (9.7%) | 2 (3.2%) | 0.2 |
| Missing | 1 | 1 | 0 |  |
| **Diabetes** | 4 (3.2%) | 3 (4.8%) | 1 (1.6%) | 0.4 |
| Missing | 1 | 1 | 0 |  |
| **Dyslipidaemia** | 5 (4.0%) | 2 (3.2%) | 3 (4.8%) | >0.9 |
| Missing | 1 | 1 | 0 |  |
| **Current/ex-smoker** | 43 (34.4%) | 21 (33.9%) | 22 (34.9%) | >0.9 |
| Missing | 1 | 1 | 0 |  |
| **CMR LV ejection fraction (%)** | 57.0 (51.0, 62.0) | 57.5 (52.5, 61.0) | 57.0 (51.0, 62.0) | 0.8 |
| Missing | 4 | 3 | 1 |  |
| **CMR LV EDV index (mL/m²)** | 88.0 (79.0, 97.5) | 92.0 (82.0, 101.0) | 84.0 (76.0, 94.0) | **0.019** |
| Missing | 22 | 16 | 6 |  |
| **CMR RV ejection fraction (%)** | 55.0 (52.0, 59.0) | 56.0 (52.0, 59.0) | 54.0 (51.0, 60.0) | 0.5 |
| Missing | 49 | 31 | 18 |  |
| **Positive T2 mapping** | 77 (88.5%) | 34 (91.9%) | 43 (86.0%) | 0.5 |
| Missing | 39 | 26 | 13 |  |
| **LGE pattern** |  |  |  | 0.4 |
| 0 | 17 (17.9%) | 9 (20.9%) | 8 (15.4%) |  |
| 1 | 67 (70.5%) | 29 (67.4%) | 38 (73.1%) |  |
| 2 | 9 (9.5%) | 3 (7.0%) | 6 (11.5%) |  |
| 3 | 2 (2.1%) | 2 (4.7%) | 0 (0.0%) |  |
| Missing | 31 | 20 | 11 |  |
| **Peak troponin** | 1,290.0 (401.0, 13,608.0) | 2,664.0 (337.0, 20,889.0) | 1,055.0 (411.0, 3,693.0) | 0.3 |
| Missing | 1 | 1 | 0 |  |
| **C-reactive protein (mg/L)** | 33.0 (9.9, 80.0) | 33.8 (10.2, 87.8) | 26.0 (7.4, 73.0) | 0.7 |
| Missing | 7 | 5 | 2 |  |
| **NT-proBNP (ng/L)** | 86.0 (63.5, 287.0) | 84.0 (60.0, 110.0) | 142.0 (72.0, 485.0) | 0.077 |
| Missing | 46 | 16 | 30 |  |
| **White cell count (×10⁹/L)** | 8.4 (6.4, 11.8) | 9.2 (6.3, 12.5) | 8.2 (6.9, 11.5) | 0.4 |
| Missing | 2 | 0 | 2 |  |
| **Admission echo LVEF (%)** | 55.0 (46.0, 58.0) | 55.0 (45.0, 58.0) | 55.0 (49.0, 59.0) | 0.4 |
| Missing | 14 | 8 | 6 |  |
| **Admission echo LVEF category** |  |  |  | 0.12 |
| Preserved (≥50%) | 75 (67.0%) | 33 (60.0%) | 42 (73.7%) |  |
| Mildly reduced (41-49%) | 20 (17.9%) | 12 (21.8%) | 8 (14.0%) |  |
| Moderately reduced (30-40%) | 13 (11.6%) | 6 (10.9%) | 7 (12.3%) |  |
| Severely reduced (<30%) | 4 (3.6%) | 4 (7.3%) | 0 (0.0%) |  |
| Missing | 14 | 8 | 6 |  |
| ^1^Median (Q1, Q3); n (%) | | | | |
| ^2^Wilcoxon rank sum test; Fisher's exact test | | | | |

Balance summary: standardised mean differences for the matching variables

| Variable | SMD before matching | SMD after matching |
| --- | --- | --- |
| Age | -0.762 | -0.007 |
| Sex | 0.112 | 0.034 |
| Propensity score | 0.653 | 0.006 |

Demographic, clinical, echocardiographic, and cardiac magnetic resonance characteristics of 126 patients (63 colchicine-treated, 63 untreated) following propensity score matching on age and sex. Continuous variables are presented as median (interquartile range) and compared using the Mann-Whitney U test; categorical variables are presented as number (percentage) and compared using Fisher’s exact test. Standardised mean differences (SMD) are reported for the variables used to estimate the propensity score, before and after 1:1 nearest-neighbour matching (caliper 0.2 SD of the logit propensity score). All absolute SMDs were below 0.1 after matching (largest 0.034), indicating adequate covariate balance.

**ACS,** acute coronary syndrome; **CMR,** cardiac magnetic resonance; **CRP,** C-reactive protein; **IQR,** interquartile range; **LGE,** late gadolinium enhancement; **LVEF,** left ventricular ejection fraction; **NT-proBNP,** N-terminal pro-B-type natriuretic peptide; **NYHA,** New York Heart Association.

**Supplementary Table 5. Clinical outcomes in the propensity score-matched cohort.**

| **Characteristic** | **Overall**  N = 126^1^ | **No colchicine**  N = 63^1^ | **Colchicine**  N = 63^1^ | **p-value**^2^ |
| --- | --- | --- | --- | --- |
| **Primary: Follow-up LVEF ≥50%** |  |  |  | 0.059 |
| *Adjusted for age and sex (Firth): OR 6.55, 95% CI 1.14 to 74.7* |  |  |  | **0.034** |
| Follow-up LVEF <50% | 8 (18.6%) | 7 (29.2%) | 1 (5.3%) |  |
| Follow-up LVEF ≥50% | 35 (81.4%) | 17 (70.8%) | 18 (94.7%) |  |
| Missing | 83 | 39 | 44 |  |
| **Secondary: all-cause mortality** |  |  |  | >0.9 |
| *Adjusted for age and sex (Firth): OR 0.61, 95% CI 0.06 to 4.67* |  |  |  | 0.63 |
| Alive | 123 (97.6%) | 61 (96.8%) | 62 (98.4%) |  |
| Death | 3 (2.4%) | 2 (3.2%) | 1 (1.6%) |  |
| **Follow-up echo LVEF (%)** | 55.0 (50.2, 58.0) | 54.0 (45.2, 57.5) | 55.2 (52.0, 58.0) | 0.2 |
| Missing | 83 | 39 | 44 |  |
| **Secondary: Absolute change in LVEF (%)** | 2.5 (-2.0, 10.0) | 1.6 (-3.0, 7.5) | 4.5 (0.0, 10.0) | 0.3 |
| Missing | 84 | 39 | 45 |  |
| **Secondary: LVEF improvement ≥10 percentage points** |  |  |  | 0.5 |
| <10 percentage point improvement | 31 (73.8%) | 19 (79.2%) | 12 (66.7%) |  |
| ≥10 percentage point improvement | 11 (26.2%) | 5 (20.8%) | 6 (33.3%) |  |
| Missing | 84 | 39 | 45 |  |
| ^1^n (%); Median (Q1, Q3) | | | | |
| ^2^Fisher's exact test; Wilcoxon rank sum test | | | | |

Event rates for the primary and secondary outcomes in 126 matched patients (63 per group). LVEF data were available for 43 patients and all-cause mortality was evaluable in all 126 patients. Binary outcomes were compared using Fisher's exact test and continuous outcomes using the Mann–Whitney U test. Adjusted odds ratios were estimated using Firth penalised logistic regression adjusted for age and sex.

**CI,** confidence interval; **LVEF,** left ventricular ejection fraction; **OR,** odds ratio.

**Supplementary Table 6 - Baseline characteristics of the propensity score-matched cohort using an expanded covariate set.**

| **Characteristic** | **Overall**  N = 106^1^ | **No colchicine**  N = 53^1^ | **Colchicine**  N = 53^1^ | **p-value**^2^ | **\|SMD\|** |
| --- | --- | --- | --- | --- | --- |
| **Age (years)** | 29.0 (23.0, 39.0) | 29.0 (22.0, 40.0) | 29.0 (26.0, 38.0) | 0.8 | 0.015 |
| **Sex** |  |  |  | >0.9 | 0.041 |
| Female | 31 (29.2%) | 15 (28.3%) | 16 (30.2%) |  |  |
| Male | 75 (70.8%) | 38 (71.7%) | 37 (69.8%) |  |  |
| **Clinical presentation** |  |  |  | >0.9 | 0.068 |
| ACS-like | 97 (91.5%) | 48 (90.6%) | 49 (92.5%) |  |  |
| Heart failure | 9 (8.5%) | 5 (9.4%) | 4 (7.5%) |  |  |
| Arrhythmia | 0 (0.0%) | 0 (0.0%) | 0 (0.0%) |  |  |
| **NYHA class** |  |  |  | 0.8 | 0.066 |
| 1 | 21 (77.8%) | 9 (81.8%) | 12 (75.0%) |  |  |
| 2 | 4 (14.8%) | 1 (9.1%) | 3 (18.8%) |  |  |
| 3 | 2 (7.4%) | 1 (9.1%) | 1 (6.3%) |  |  |
| Missing | 79 | 42 | 37 |  |  |
| **Chest pain** | 92 (86.8%) | 40 (75.5%) | 52 (98.1%) | <0.001 | 0.710 |
| **Dyspnoea** | 34 (32.1%) | 14 (26.4%) | 20 (37.7%) | 0.3 | 0.244 |
| **Syncope** | 1 (0.9%) | 0 (0.0%) | 1 (1.9%) | >0.9 | 0.196 |
| **ST elevation (≥2 mm in ≥2 leads)** | 33 (34.4%) | 14 (28.0%) | 19 (41.3%) | 0.2 | 0.282 |
| Missing | 10 | 3 | 7 |  |  |
| **Hypertension** | 4 (3.8%) | 2 (3.8%) | 2 (3.8%) | >0.9 | 0.000 |
| **Diabetes** | 3 (2.8%) | 2 (3.8%) | 1 (1.9%) | >0.9 | 0.114 |
| **Dyslipidaemia** | 5 (4.7%) | 2 (3.8%) | 3 (5.7%) | >0.9 | 0.089 |
| **Current/ex-smoker** | 42 (39.6%) | 20 (37.7%) | 22 (41.5%) | 0.8 | 0.077 |
| **CMR LV ejection fraction (%)** | 57.0 (51.0, 62.0) | 58.0 (54.0, 64.0) | 56.0 (51.0, 60.0) | 0.10 | 0.164 |
| Missing | 3 | 2 | 1 |  |  |
| **CMR LV EDV index (mL/m²)** | 86.5 (76.0, 98.0) | 90.5 (78.0, 100.0) | 83.5 (75.0, 94.0) | 0.2 | 0.171 |
| Missing | 16 | 11 | 5 |  |  |
| **CMR RV ejection fraction (%)** | 56.0 (53.0, 60.0) | 56.0 (53.0, 59.0) | 54.0 (52.0, 60.0) | 0.4 | 0.180 |
| Missing | 42 | 26 | 16 |  |  |
| **Positive T2 mapping** | 67 (90.5%) | 31 (100.0%) | 36 (83.7%) | 0.037 | 0.624 |
| Missing | 32 | 22 | 10 |  |  |
| **LGE pattern** |  |  |  | 0.3 | 0.420 |
| 0 | 19 (22.4%) | 12 (29.3%) | 7 (15.9%) |  |  |
| 1 | 57 (67.1%) | 24 (58.5%) | 33 (75.0%) |  |  |
| 2 | 8 (9.4%) | 4 (9.8%) | 4 (9.1%) |  |  |
| 3 | 1 (1.2%) | 1 (2.4%) | 0 (0.0%) |  |  |
| Missing | 21 | 12 | 9 |  |  |
| **Peak troponin** | 1,600.0 (411.0, 19,893.0) | 3,117.0 (426.0, 21,193.0) | 1,099.0 (411.0, 7,585.0) | 0.3 | 0.252 |
| **C-reactive protein (mg/L)** | 34.5 (12.4, 80.8) | 34.4 (13.8, 85.0) | 38.5 (10.0, 80.0) | 0.8 | 0.047 |
| **NT-proBNP (ng/L)** | 86.5 (65.0, 233.0) | 83.0 (61.0, 103.0) | 167.0 (69.0, 605.0) | 0.054 | 0.010 |
| Missing | 40 | 14 | 26 |  |  |
| **White cell count (×10⁹/L)** | 9.2 (6.9, 11.6) | 9.4 (6.9, 10.8) | 8.2 (6.9, 11.8) | 0.6 | 0.070 |
| Missing | 2 | 0 | 2 |  |  |
| **Admission echo LVEF (%)** | 55.0 (47.0, 58.0) | 55.0 (46.0, 58.0) | 55.0 (48.0, 59.0) | >0.9 | 0.095 |
| **Admission echo LVEF category** |  |  |  | 0.5 | 0.348 |
| Preserved (≥50%) | 74 (69.8%) | 36 (67.9%) | 38 (71.7%) |  |  |
| Mildly reduced (41-49%) | 15 (14.2%) | 7 (13.2%) | 8 (15.1%) |  |  |
| Moderately reduced (30-40%) | 14 (13.2%) | 7 (13.2%) | 7 (13.2%) |  |  |
| Severely reduced (<30%) | 3 (2.8%) | 3 (5.7%) | 0 (0.0%) |  |  |
| ^1^Median (Q1, Q3); n (%) | | | | | |
| ^2^Wilcoxon rank sum test; Fisher's exact test | | | | | |

Cohort after 1:1 nearest-neighbour propensity score matching on the expanded covariate set (age, sex, clinical presentation, admission echo LVEF, hypertension, log CRP, log peak troponin), caliper 0.2 SD of the logit propensity score, seed 20260531. Matching was performed among the 198 patients with complete data on all seven covariates (57 colchicine, 141 untreated), yielding 106 matched patients (53 per group); 4 colchicine-treated patients could not be matched within the caliper. Values are median (Q1, Q3) or n (%); |SMD| is the absolute standardised mean difference between groups after matching. The largest |SMD| across all tabulated characteristics was 0.710.

**Supplementary Table 7 - Sensitivity analysis of patients with and without follow-up echocardiography**

| Characteristic | Available (n=93) | Not available (n=150) | \|SMD\| full cohort | \|SMD\| colchicine arm | \|SMD\| untreated arm |
| --- | --- | --- | --- | --- | --- |
| Age (years), median (Q1, Q3) | 42.0 (28.0, 55.0) | 32.0 (26.0, 48.0) | 0.334 | 0.391 | 0.286 |
| Male sex, n (%) | 54 (58.1%) | 106 (71.1%) | 0.276 | 0.535 | 0.184 |
| Heart failure presentation, n (%) | 22 (23.9%) | 13 (8.8%) | 0.519 | 0.228 | 0.550 |
| Admission echo LVEF (%), median (Q1, Q3) | 47.5 (39.0, 58.0) | 55.0 (50.2, 58.0) | 0.605 | 0.604 | 0.576 |
| CMR LVEF (%), median (Q1, Q3) | 56.0 (49.0, 62.0) | 58.0 (53.5, 63.0) | 0.398 | 0.305 | 0.435 |
| Chest pain, n (%) | 61 (65.6%) | 127 (86.4%) | 0.502 | 0.333 | 0.503 |

Sensitivity analysis addressing whether the primary outcome data are missing at random. An additional 16 baseline characteristics were compared (comorbidities, laboratory values, LGE pattern, remaining presentation subcategories and NYHA class). Of these, 2 exceeded a standardised mean difference of 0.3 in the full cohort comparison (LGE pattern, 0.52; white cell count, 0.37), and the remaining 14 were all below 0.3; none is tabulated here. Follow-up LVEF was available in 19 of 63 colchicine-treated patients (30.2%) and 74 of 178 untreated patients (41.6%; SMD 0.240). 5 additional patients had a follow-up echocardiogram without a recorded ejection fraction and were treated as missing for the primary outcome.

**Supplementary Table 8. Sensitivity analyses of the colchicine treatment effect for LVEF recovery to ≥50% using expanded propensity score models.**

| **Covariate set** | **Method** | **n** | **OR** | **95% CI** | **p** |
| --- | --- | --- | --- | --- | --- |
| 7 covariates | 1:1 matching | 40 | 5.00 | 0.93–51.31 | 0.061 |
| 7 covariates | IPTW | 88 | 4.34 | 0.50–37.37 | 0.179 |
| **7 covariates** | **PS as covariate** | **88** | **4.67** | **1.03–44.73** | **0.046** |
| 8 covariates (+CMR LVEF) | 1:1 matching | 39 | 3.89 | 0.67–40.94 | 0.134 |
| 8 covariates | IPTW | 84 | 4.87 | 0.56–41.99 | 0.148 |
| **8 covariates** | **PS as covariate** | **84** | **4.86** | **1.07–46.53** | **0.040** |

The seven-covariate model estimated the propensity score from age, sex, clinical presentation, admission echocardiographic LVEF, hypertension, log C-reactive protein (CRP), and log peak troponin (complete-case n=198 of 241; 106 matched, 53 per group, 4 colchicine-treated patients unmatched within the caliper). The eight-covariate model additionally incorporated cardiac magnetic resonance (CMR) LVEF (complete-case n=190 of 241, 56 colchicine-treated and 134 untreated; 106 matched, 53 per group, 3 colchicine-treated patients unmatched). Late gadolinium enhancement pattern and N-terminal pro-B-type natriuretic peptide (NT-proBNP) were not included in either model, as these were missing in 28% and 34% of patients, respectively. Each covariate set was applied via three methods: 1:1 nearest-neighbour matching without replacement (caliper 0.2 SD of the logit propensity score); inverse probability of treatment weighting (IPTW) with stabilised weights truncated at the 1st and 99th percentiles; and inclusion of the logit propensity score as a covariate alongside colchicine treatment in a Firth penalised logistic regression outcome model. Treatment effects are reported for LVEF recovery to ≥50% at follow-up echocardiography.

***CI,*** *confidence interval;* ***CMR,*** *cardiac magnetic resonance;* ***IPTW,*** *inverse probability of treatment weighting;* ***LVEF,*** *left ventricular ejection fraction;* ***OR,*** *odds ratio;* ***PS,*** *propensity score.*
